# Response to Chagas disease in Brazil: strategic milestones for achieving comprehensive health care

**DOI:** 10.1590/0037-8682-0193-2022

**Published:** 2022-04-29

**Authors:** Alberto Novaes Ramos, Eliana Amorim de Souza, Maria Cristina Soares Guimarães, Debbie Vermeij, Marly Marques Cruz, Alejandro O. Luquetti, Liléia Diotaiuti, Swamy Lima Palmeira, Mayara Maia Lima, Veruska Maia da Costa, Luiz Antonio Botelho Andrade, Dalmo Correia, Andréa Silvestre de Sousa

**Affiliations:** 1 Universidade Federal do Ceará, Faculdade de Medicina, Programa de Pós-Graduação em Saúde Pública, Fortaleza, CE, Brasil.; 2 Universidade Federal do Ceará, Faculdade de Medicina, Departamento de Saúde Comunitária, Fortaleza, CE, Brasil.; 3 Universidade Federal da Bahia, Instituto Multidisciplinar em Saúde, Núcleo Epidemiologia e Saúde Coletiva, Vitória da Conquista, BA, Brasil.; 4 Fundação Oswaldo Cruz, Escola Nacional de Saúde Pública Sergio Arouca, Rio de Janeiro, RJ, Brasil.; 5 Fundação Oswaldo Cruz, Instituto de Comunicação e Informação Científica e Tecnológica em Saúde, Rio de Janeiro, RJ, Brasil.; 6 Fundação Oswaldo Cruz, Instituto Nacional de Infectologia Evandro Chagas, Rio de Janeiro, RJ, Brasil.; 7 Universidade Federal de Goiás, Hospital das Clínicas, Núcleo de Estudos da Doença de Chagas, Goiânia, GO, Brasil.; 8 Fundação Oswaldo Cruz, Instituto René Rachou, Grupo de Pesquisa Triatomíneos, Belo Horizonte, MG, Brasil.; 9 Ministério da Saúde, Secretaria de Vigilância em Saúde, Brasília, DF, Brasil.; 10 Universidade Federal Fluminense, Instituto de Biologia, Departamento de Imunobiologia, Niterói, RJ, Brasil.; 11 Universidade Federal de Sergipe, Curso de Pós-Graduação em Ciências da Saúde, Aracaju, SE, Brasil.; 12 Universidade Federal do Rio de Janeiro, Faculdade de Medicina, Departamento de Clínica Médica, Rio de Janeiro, RJ, Brasil.

Chagas disease (CD) is a complicated, neglected, and muted disease that, in the 21^st^ century, is increasingly taking on global contours, affecting populations on nearly every continent. 

In 2019, during the 72^nd^ World Health Assembly, the World Health Organization (WHO) designated April 14 as World Chagas Disease Day (https://www.who.int/campaigns/world-chagas-disease-day/2021). The establishment of this day is a result of the international community’s efforts to seek increased visibility of not only CD itself but also the individuals it affects^1^ ^,^ ^2^. 

World Chagas Disease Day reinforces the current challenges and future agendas for the disease by highlighting the need to overcome several common barriers that limit individuals affected by CD to access effective and efficient diagnosis, treatment, and aftercare^2^ ^-^ ^4^. There are critical problems in achieving disease control that have been recognized, such as the lack of verified data on the burden of CD; the limitation of integrated surveillance, control, and care at the primary health care (PHC) level; the geographic distance of individuals affected by CD from health services; a complex, frequently cumbersome, time-consuming, and expensive diagnostic process; a lack of integration with reproductive, maternal, newborn, and child health policies and practices; a disproportionate impact of the disease in vulnerable populations; limited level of knowledge about CD in both the general population and health professionals; limited media attention; limited health education initiatives; limited availability of related tools and materials in health centers; fear; stigma and discrimination against individuals affected by CD; low social mobilization; and a limited political voice of individuals at risk of contracting CD^1^ ^-^ ^4^.

The importance of this initiative becomes even more apparent when considering that approximately 90% of the individuals affected by CD do not have an established diagnosis and that only 1% of those who have been diagnosed with CD undergo etiological treatment^2^ ^-^ ^4^. This contributes to the high morbidity and mortality from CD, as well as its substantial social and economic impact^4^ ^,^ ^5^. Therefore, we reiterate the importance of implementing major strategic benchmarks for the control of neglected tropical diseases (NTDs), a group of diseases in which CD was designated in 2021 through the WHO roadmap for NTDs 2021-2030, particularly from a One Health perspective^5^. One Health is a relevant and holistic approach to attain Sustainable Development Goals and to develop more effective and evidence-based actions to be implemented at the local, regional, national, and global health levels^4^ ^-^ ^6^. There is a continuous and clear need for integrated surveillance, control, and health care actions considering the principles of the One Health approach for controlling CD and NTDs, from a transdisciplinary perspective^6^.

Since the discovery of CD by Carlos Chagas, Brazil has sought to build a framework of policies and strategies for planning, managing, and implementing health care services dedicated to CD, in addition to enabling strategic research and prioritizing health surveillance, particularly in the entomological and epidemiological aspects during the acute phase of CD^2^.

We have come a long way, but much progress is still needed, especially in the integrated actions between the government and civil society.

In the 1970s and 1980s, the Brazilian Ministry of Health (MoH) initiated studies, for which the Brazilian Society of Tropical Medicine (BSTM) had a strong presence, to estimate the prevalence of CD in the country^4^. In the 1990s, the MoH participated in a joint initiative with other Southern Cone governments, which culminated in 2006 with the International Certification of Elimination of Transmission of CD by *Triatoma infestans* in Brazil, a significant milestone^4^ ^,^ ^7^. The entomological surveillance monitoring for identifying native triatomine species with a potential risk of domiciliation should be intensified^8^. To assess the impact of vector control on the transmission of the disease, a study using a new national seroprevalence survey was conducted in Brazil from 2001 to 2008, demonstrating the success of the efforts^4^ ^,^ ^7^ ^,^ ^9^. 

However, the risk for vector-borne transmission by secondary triatomine species that are typical of Brazilian ecosystems, has persistently occurred^2^ ^,^ ^8^. The reduction of *Trypanosoma cruzi* vector-borne and transfusion-borne transmission increased the visibility of other, less frequently occurring modalities, such as oral transmission and vertical transmission, which, until then, were considered to have minor relevance^4^ ^,^ ^7^ ^,^ ^9^ but have since become increasingly important. This has faced the Brazilian surveillance approach with new challenges: the much-needed integration between health surveillance and maternal and child health care services and the growing concern of chronic CD^2^ ^,^ ^4^, the most silent and unspoken dimension of CD.

The first Brazilian Consensus on CD, a partnership between the MoH and BSTM in 2005, enabled the standardization of procedures for diagnosing and treating the disease in the acute and chronic phases, indicating the potential expansion of the criteria used for antiparasitic treatment^10^. *T. cruzi* and human immunodeficiency virus (HIV) co-infection was also widely discussed then, which resulted in the development of guidelines for the surveillance and management of co-infection and the treatment of reactivated CD^2^ ^,^ ^10^. We also highlight the relevant role of the Brazilian Society of Cardiology and its publication of the First Latin American Guidelines for the Diagnosis and Treatment of Chagasic Cardiopathy in 2011, which discussed the most prevalent symptomatic clinical form of CD as having a high burden of morbidity and mortality^11^.

Debates regarding the diagnostic and treatment protocols for CD in Brazil continued until 2015 when the revised Brazilian Consensus on CD was published. The propositions included the necessary debate on the potential indications for antiparasitic treatment and the need for evaluating new diagnostic tools, such as rapid screening tests for *T. cruzi* infection^2^. A consensus was critical for inducing movements for the mobilization of the National Commission for the Incorporation of Technologies in the Unified Health System (SUS) for preparing the Clinical Protocol and Therapeutic Guidelines (PCDT) for CD, which were eventually published in 2018^12^. The PCDT reiterated the importance of determining the actual number of individuals affected by CD and advocated for chronic CD surveillance and the actions necessary for ensuring that these individuals have access to diagnostic and treatment services^4^ ^,^ ^7^ ^,^ ^12^, moving toward comprehensive care within the guiding principles of the SUS. The recommendations for incorporating screening programs focusing on both the undetermined chronic form of CD and CD in pregnant women in the context of their high risk and vulnerability were notable.

Another key step was taken in 2020 when the MoH implemented an ordinance that standardized the notification of chronic cases throughout the country^13^. Efforts are now being directed toward structuring the surveillance methods, starting with the implementation of compulsory notifications in the SUS information system. 

Amid the advances previously reported, national health systems face new challenges, particularly the emergence of severe acute respiratory syndrome coronavirus 2 (SARS-CoV-2) and the coronavirus disease 2019 (COVID-19) in 2020^13^. MoH analysis indicate an increasing trend in the morbidity and mortality from CD, as well as a reduction in the surveillance and control efforts in the country^13^, adding to the challenges that need to be overcome.

Another important dimension of the path to achieving comprehensive care for CD is the development of a line of care, in addition to the structuring of mortality surveillance mechanisms^13^. Proposals to structure and implement a framework for promoting comprehensive care, surveillance, and other actions in prioritized territories have been developed^13^ ^,^ ^14^. 

In 2020, the Pan American Health Organization published the document titled “Chronic care for neglected infectious diseases: leprosy, lymphatic filariasis, trachoma, and Chagas disease-A guide for morbidity management and disability prevention for primary health care services”^15^. In this document, the contribution of Prof. João Carlos Pinto Dias synthesized the voice of several Brazilian researchers who reinforced the important role of PHC in the management of CD for those affected by the disease. It highlighted the importance of the integration of PHC with surveillance actions in territories in which they operate^15^. This integration reinforces the importance of utilizing information, education, and communication (IEC) strategies, which have not been widely included in CD control programs. Moreover, programs face intersectorial challenges, including failures in science, the market, and the health system, as well as the need to overcome poverty, which requires a holistic response based on human and social development strategies^4^ ^-^ ^7^. Integra-Neglected Diseases (Integra-DN), an initiative aimed at integrating NTD-related health surveillance with PHC services, is one of the first initiatives with this goal. Integra-DN was developed in 2018 as the result of a partnership between the MoH and the Institute of Communication and Scientific and Technological Information in Health. It includes the participation of experts from various fields, including those from fields related to CD. Integra-DN aimed to implement practices that extended beyond standard practices and focused on taking a broad look at neglected populations and territories, seeking to mobilize PHC and surveillance professionals around potential spaces to facilitate local action and achieve comprehensive care.

More recently, Brazil has redeemed its role as a leader in implementing strategic projects from both national and international perspectives^13^. Through two large projects, the country is committed to expanding and ensuring PHC access to screening and treatment services for CD while ensuring the integration of PHC with surveillance^13^ and international guidelines^16^ to increase the possibility of curing or preventing the disease from evolving into more severe clinical forms.

In 2019, the pilot project titled “Access to the detection and treatment of CD in the context of primary healthcare in Brazil - IntegraChagas”^13^ was implemented. Coordinated by the National Institute of Infectology Evandro Chagas (INI/Fiocruz) in partnership with the Federal University of Ceará, IntegraChagas Brazil includes strategies aimed at formulating permanent education, IEC strategies, and community-based actions. The institutions involved in the implementation and operation projects are the MoH; the technical and scientific units of the Fiocruz, State, and Municipal Health Secretariats of municipalities in the States of Goiás, Bahia, Pernambuco, Minas Gerais, and Pará (the latter in a specific arm); social movements; and non-governmental institutions. This wide range of partnerships is essential for the project to succeed. 

On April 14, 2021, the symbolic signing of the contract for the “Communities United for Innovation, Development, and Care for Chagas Disease” project, a partnership between the MoH, Fiocruz/Fiotec, and Unitaid, took place. This project is focused on eliminating the mother-to-child transmission of CD. It includes research designed to provide replicable and adaptable implementation models for different contexts, as well as innovative strategies for improving diagnostic services (with new tools, such as rapid testing, in the SUS) and treatment options (with new therapeutic schemes). In partnership with the governments of Bolivia, Brazil, Colombia, and Paraguay, the project is expected to reach 234,000 individuals, including women of childbearing age, their children, and their household contacts^13^.

Around the same time, Brazil signed an agreement to participate in the “Ibero-American Initiative on Congenital Chagas” (“No baby with Chagas, the path to new generations without Chagas”), which was constituted in 2021 during the XXVII Ibero-American Summit of Heads of State and Government^13^. In partnership with the INI/Fiocruz in Brazil, Argentina, Colombia, Spain, El Salvador, Honduras, Guatemala, and Paraguay have a shared objective of contributing to the elimination of mother-to-child transmission of Chagas disease from a multidimensional approach, taking into account control and prevention strategies for other forms of transmission.

These collaborative efforts and movements to combat CD have been fostered by and reinforced through social participation. The Brazilian Social Forum to Combat Infectious and Neglected Diseases^4^ joined the International Federation of Associations of People Affected by CD (FINDECHAGAS), which includes individuals and communities affected by CD in endemic and non-endemic countries^2^. One of the initiatives developed from this movement is the leadership training course, which is based on the belief that local problems are addressed through the implementation of local solutions. By capacitating local representatives and leaders, those who are often overlooked are given a voice and can better facilitate change. 

Effective and efficient solutions to the many challenges facing those affected by CD and providing hope and comprehensive care to those that need it, the most can be achieved only through collective efforts by governments, knowledge-based institutions, civil society, communities, and donors.
